# The dark side of herbivorous insects in Neotropical savannas: plant–herbivore networks across diurnal and nocturnal periods

**DOI:** 10.1007/s00442-026-05996-1

**Published:** 2026-10-27

**Authors:** Jefferson Bruno B. Souza-Oliveira, Leonardo V. A. Costa, Wesley Dáttilo, Walter S. Araújo

**Affiliations:** 1 Laboratório de Interações Ecológicas E Biodiversidade, Programa de Pós-Graduação Em Biodiversidade E Uso Dos Recursos Naturais, Departamento de Biologia Geral, Centro de Ciências Biológicas E da Saúde, Universidade Estadual de Montes Claros, Montes Claros, MG Brazil; 2 Red de Ecoetología, Instituto de Ecología AC, Xalapa, Veracruz, Mexico; 3 Tecnología E Innovación, Laboratorio Nacional de Biología del Cambio Climático, Secretaría de Ciencia, HumanidadesMexico City, Mexico

**Keywords:** Exophagous insects, Herbivory patterns, Insect activity, Insect behavioral, Temporal dynamics

## Abstract

Herbivorous insects exhibit temporal plasticity in their activity, particularly under high diurnal stress, with many groups shifting to nighttime activity due to ecophysiological constraints. However, how their interaction organization changes across dsaily time periods remains poorly understood. We investigated structural patterns in plant–herbivore networks composed of free-living insects across diurnal, nocturnal, and complete (day + night) networks in Neotropical savannas. Additionally, we assessed changes in insect species richness, network turnover and rewiring, and species roles. Here, we sampled plant–herbivore interactions and constructed ecological networks, with interaction weights based on the abundance of herbivorous species recorded on sampled plants. Insect species richness was similar between nocturnal and complete networks, and both showed twice the richness observed in diurnal networks. Overall dissimilarity between diurnal and nocturnal networks was 67%, with 56% attributed to interaction rewiring and 43% to species turnover. Species roles shifted across periods: six species (including insects and plants) served as network hubs during the day, whereas five species were hubs at night, with only one insect and one plant species serving as hubs across periods. Nevertheless, all networks remained more specialized, modular, and weakly nested — as expected by chance — and maintained similar structures across periods. Our findings indicate that herbivorous insects in our sampled Neotropical savanna respond to diel changes, with nighttime representing an important period for their occurrence and interaction roles. However, the network structure remains robust to these changes, suggesting that species across periods may exhibit functional redundancy that maintains network structure.

**Supplementary Information:**

The online version contains supplementary material available at https://doi.org/10.1007/s00442-026-05996-1.

## Introduction

Plant–herbivore interactions are essential for transferring energy across trophic levels, modulating habitat structure, and driving the adaptation and diversification of herbivorous species and their hosts (Basset et al. 2012; Wijas et al. 2023). Consequently, the behavioral patterns of herbivorous species play a key role in their fitness (Gripenberg et al. 2010; Myers and Sarfraz 2017). For example, exophagous herbivorous insects exhibit greater interaction plasticity, resulting in a higher host-switching capacity and, consequently, more generalist patterns than endophagous insects (Oliveira et al. 2019; Silveira and Araújo 2021). Additionally, they exhibit a broader spatial distribution across their habitats, contributing to pronounced turnover in species and their interactions (Martins et al. 2019). These structural patterns support more flexible foraging strategies because movement and foraging are less restricted (Price 2002; Neves et al. 2010). Nonetheless, exophagous herbivores still exhibit host preference, interacting with phylogenetically related plant species (Ødegaard et al. 2005; Castagneyrol et al. 2013). Taken together, these contrasting patterns highlight the central role of interaction plasticity and host specificity in organizing plant–insect interactions (Miranda et al. 2026). This behavioral flexibility extends across temporal scales, enabling herbivores to partition resources not only spatially but also temporally, shifting activity patterns in response to environmental pressures (Kronfeld-Schor and Dayan 2003).

Habitat use is structured by both biotic and abiotic factors that modulate behavior and interaction patterns (Hassell and Southwood 1978). Ultimately, adaptive plasticity enables behavioral changes that lead to resource partitioning among species in the same habitat (Powell et al. 2024). Consequently, shifts in behavioral patterns may be crucial for optimal habitat use by herbivorous insects and for promoting their persistence in heterogeneous environments (Scherrer et al. 2016). Temporal partitioning, particularly shifts between diurnal and nocturnal activity, is a key mechanism for resource partitioning among herbivorous species in the same habitat (Kronfeld-Schor and Dayan 2003; Scherrer et al. 2016; Powell et al. 2024). For example, day-night partitioning facilitates access to different resources in response to environmental pressures (Kronfeld-Schor and Dayan 2003; Scherrer et al. 2016). Extreme daytime conditions, such as high temperatures, low humidity, and the release of concentrated plant defensive volatiles during intense light periods (Ballaré 2014; de Vries et al. 2017; Lazzarin et al. 2021), may limit insect activity (Wong and Didham 2024). However, nocturnal activity is an escape mechanism from extreme daytime conditions in dry environments (Janzen 1973; Silva et al. 2017), leading to a high occurrence of herbivorous insects in their hosts at night (Dáttilo et al. 2015).

Tropical ecosystems represent the world’s major biodiversity hotspots, characterized by high insect diversity and a broader range of habitats and resources, which contribute to the diversification of species interactions (Raimundo et al. 2018). Nevertheless, tropical ecosystems are highly dynamic, leading to temporal variation in species’ interactions (Leal et al. 2016; Flores-Flores et al. 2018; López-Carretero et al. 2018). For example, the Brazilian Cerrado is a challenging environment for free-living herbivorous insects, which allocate several mechanisms to modulate their ecological patterns (e.g., Neves et al. 2010). This Neotropical savanna exhibits well-defined seasons, with daily periods of extreme temperatures and humidity stress, sclerophyllous vegetation, diverse plant defense traits, and specialized predators (Moraes et al. 1999; da Silva and Batalha 2011; Araújo et al. 2024). These conditions likely drive pronounced temporal niche partitioning between diurnal and nocturnal herbivore assemblages. Nocturnal herbivores, operating under more stable conditions, are expected to exhibit higher specialization in their plant interactions. In contrast, diurnal herbivores, constrained by extreme conditions, are expected to show broader host ranges. Although our knowledge of the ecological patterns of plant–herbivore interactions in the Cerrado is typically addressed spatially, temporal divisions are often limited to seasonality or diurnal partitioning (e.g., Oliveira et al. 2019; Araújo and Oliveira 2021; Araújo et al. 2024), leaving nighttime gaps to be addressed.

Interaction networks are ecological representations of the structural organization of species interactions and serve as pivotal tools for understanding key ecological patterns in plant-animal interactions (Lewinsohn et al. 2006; Almeida-Neto et al. 2008; López-Carretero et al. 2018), with broader applications in tropical habitats (Moreira et al. 2018; Vizentin-Bugoni et al. 2018; Oliveira et al. 2024). When herbivorous insects exhibit correlated host preferences, plant–herbivore networks exhibit restricted structural organization, with specialized, modular interactions and low nestedness (Oliveira et al. 2024). Even so, whether these ecological patterns extend into nighttime remains unclear, as broader studies focus on other insect systems such as ants or bees (Dáttilo et al. 2014; Luna et al. 2018; Ratoni et al. 2025; Miranda et al. 2026). Although plant communities remain constant across diel cycles, herbivore assemblage composition shifts between day and night, potentially rewiring interaction patterns. Consequently, we expect high dissimilarity in plant–herbivore networks between periods, with different herbivore species occupying distinct functional roles (such as specialists or network hubs) reflecting shifts in ecological roles within the same plant community.

Here, we investigate how interaction patterns between free-living herbivorous insects and their host plants are structured across day and night in Brazilian savannas. To address this question, we measured the structure of plant–herbivore interaction networks during daytime and nighttime. We also evaluated changes in herbivorous insect species and their interactions with plants between both periods. To this end, we quantified the temporal turnover of herbivorous insect species and the temporal rewiring of their interactions across daytime, nighttime, and complete periods (day + night). To complement this, we assessed potential shifts in species roles across periods to provide a comprehensive description of network changes. Given the distinct environmental conditions between day and night in the Cerrado, we expect: (i) a high dissimilarity between networks driven by species turnover and interaction rewiring, because herbivore assemblage composition shifts between day and night, despite constant plant communities. Additionally, (ii) shifts in species functional roles across networks, with different herbivore species occupying distinct roles, such as specialists or network hubs, between periods. Given the patterns of specialization in plant–herbivore interactions, we also expect that (iii) daytime and nighttime networks are naturally characterized by specialized topologies. However, given stable environmental conditions at night, we expect that (iv) nighttime plant–herbivore networks are more specialized than daytime networks.

## Materials and methods

### Study area

We conducted the study at the Reserva Particular de Patrimônio Natural Porto Cajueiro (RPPN Porto Cajueiro), located in the municipality of Januária, northern Minas Gerais, Brazil (14°53'18.28"S/45°20'30.75"W). Covering approximately 20,000 ha, the reserve is part of the *Sertão Veredas–Peruaçu Mosaic*, a set of protected areas along the São Francisco River. The RPPN is dominated by Cerrado *stricto *sensu (neotropical savanna), characterized by shrub vegetation and small trees, with an Aw climate (Köppen classification), a mean annual temperature of 22 °C, and a mean annual precipitation of 1,000 mm, with a well-defined dry season during winter (Alvares et al. 2014; Silva et al. 2024).

### Herbivore-plant sampling

To sample plant–herbivore interactions, we used a phytocentric approach that captures insect herbivores on their host plants (e.g., Oliveira et al. 2019). We selected two sites along an upstream–downstream gradient, each separated by two km and located near *veredas* (palm swamp forest) phytophysiognomy. At each site, we established five 100 m^2^ plots (10 × 10 m), spaced at least 20 m apart, for a total of 10 plots. Within each plot, we marked all living woody plants (trees and shrubs) with a circumference at breast height (CBH, 1.30 m above ground) ≥ 15 cm. For each marked plant individual, we recorded CBH and total height and identified specimens in the field to the lowest possible taxonomic level using field guides and expert assistance. We collected unidentified individuals for further identification using specialized publications and/or consultation with laboratory taxonomic experts.

Insect herbivores were sampled from host plants using the beating method (Oliveira et al. 2019) during both daytime (from 08:00 a.m. to 5:00 p.m.) and nighttime (from 06:00 p.m. to 12:00 a.m.) in all plots (10 sampling events per period) on the same day. For each plant sampled, we arbitrarily selected three branches and performed 10 beats per branch with a wooden stick, causing arthropods to fall onto a white cloth (100 × 100 cm). We resampled the same individual plants in each plot at night. During nocturnal sampling, we used red-light flashlights to avoid attracting additional insects to the sampled plants. We collected and preserved all insect specimens in 70% alcohol and recorded plot, site, and host plant information. In the laboratory, we separated insect individuals into morphospecies, identified them to the lowest possible taxonomic level and classified them as insect herbivores using identification keys from the literature (Rafael et al. 2024).

### Plant–herbivore networks

For analyses, we pooled data from all sampling plots within each sampling period (daytime, nighttime, and combined). We excluded interactions involving immature insect herbivores, as they have less flexibility to switch host plants than adults, which could bias our data (e.g., Silveira and Araújo 2021). We then constructed one weighted interaction matrix for the daytime period, one for the nighttime period, and a third matrix combining both periods (day + night). The matrices represent bipartite network data, where columns correspond to insect herbivore species, rows to host plants and cell values indicate the frequency of interactions between them. Interaction weights were based on the abundance of herbivorous insect species recorded on each host plant in each dataset (daytime, nighttime, and combined). We therefore obtained a single general diurnal, nocturnal, and complete matrix, yielding three general plant–herbivore networks.

### Herbivorous insect richness

To assess insect herbivore richness across plant–herbivore networks, we plotted rarefaction curves based on species abundance, standardizing sample size by the number of individuals across periods. We used the iNEXT package to construct rarefaction curves (Chao et al. 2014). Hill numbers allow fair quantification and comparison of species diversity while controlling for differences in sampling effort (Hsieh and Chao 2016). We employed species richness (q = 0), which reflects the number of species without considering their relative abundances, giving equal weight to rare and abundant species. We computed rarefaction curves with 95% confidence intervals. We extrapolated to three times the observed sample size of the treatment with the lowest insect abundance. Differences between environments are not expected when intervals overlap (Chao et al. 2014).

### Network turnover and rewiring

To assess herbivore turnover and interaction rewiring within plant–herbivore networks, we applied the interaction beta-diversity framework (Poisot et al. 2012; Legendre 2014; CaraDonna et al. 2017), which quantifies total dissimilarity between networks (β*wn*) and partitions it into two main components. First, turnover (β*st*) refers to changes in networks due to species turnover (species entering or leaving). For instance, if herbivorous insect species disappear from a network, all of their interactions disappear because of their absence, not because of changes in their partners. Additionally, rewiring (β*rw* or β*o*) refers to the reorganization of interactions or partner switching among species shared between networks. For example, when the same herbivorous insect species is present in two networks but interacts with different host plants, this contributes to rewiring. Thus, the overall dissimilarity between networks is composed of these two components:

$$\beta w=\frac{a+b+c}{(2a+b+c)}-1$$

where *a* is the number of species or interactions shared between networks, *b* is the number of species or interactions unique to the first network, and *c* is the number of species or interactions unique to the second network. These measures range from 0 (no dissimilarity) to 1 (complete dissimilarity) (Luna et al. 2020). To evaluate temporal beta diversity of interactions, we calculated Bray–Curtis dissimilarity between diurnal and nocturnal networks (pairwise comparisons between networks from both periods). For the beta-diversity analysis, we used the *betalinkr* function to assess temporal differences between diurnal and nocturnal networks.

### Network species role

In addition, we assessed whether insect herbivores and plant species acted as peripheral (few interactions) or network hubs (highly connected species with strong importance for overall connectivity). To describe hub and peripheral species in the networks, we used the following formula:

$$Gc=\frac{ki-kmean}{\sigma k}$$

where *k*_*i*_ is the number of links for a given herbivore or plant species, *k*_*mean*_​ is the average number of links for all herbivore or plant species in the network, and σ_*k*_ is the standard deviation of links among all species. Species with Gc ≥ 1 are considered hub species, while those with Gc ​< 1 are considered peripheral. In other words, Gc​ is a standardized measure quantifying how much a species’ number of interactions deviates from the network interaction mean (Dáttilo et al. 2013, 2014).

### Network structure

To evaluate changes in network structure across day and night, we extracted different topological descriptors, including specialization, nestedness, and modularity, for the general diurnal, nocturnal and complete networks. We measured network specialization using the *H*^*2*^*’* index, which assesses species’ interaction preferences based on the weight of their interactions relative to the number of available partners in the network. *H*^*2*^*’* ranges from 0, indicating a completely generalized network, to 1, indicating a completely specialized one. Nestedness was calculated using the *WNODF* metric (weighted nestedness). Nestedness describes a pattern in which generalist species (e.g., herbivores) interact strongly with other generalists (e.g., plants) as well as with specialists, which rarely interact with one another. In other words, specialist herbivores tend to interact with a subset of generalist plants, which themselves interact with both generalist and specialist herbivores. Nestedness ranges from 0 for a weakly nested network to 100 for a perfectly nested one (Almeida-Neto et al. 2008). Finally, we calculated modularity using the *DIRTLPAwb*+ algorithm (Beckett 2016). Modularity quantifies the formation of subgroups within the network, in which species interact more frequently among themselves than with the rest of the network, thereby forming distinct interaction modules. Modularity ranges from 0 (non-modular network) to 1 (highly modular network).

To assess whether the measured network patterns differed from random expectations, we used null models to compare them with the observed networks (day, night, and complete). We generated 1,000 null networks using the *r2dtable* algorithm, which preserves the marginal totals (abundance/frequency of interactions) of the randomized networks identical to those observed. Because network patterns are influenced by species abundance, the *r2dtable* algorithm allows us to isolate the structure that emerges in null networks beyond this effect. If a network metric differs significantly from the distribution generated by *r2dtable*, this provides evidence that non-random organizational processes are at play (e.g., interaction preferences) (Dormann et al. 2025).

For each network pair, we assessed whether specialization and modularity differed between periods using paired permutation tests against null models (e.g., Anjos et al. 2018). For each comparison, we first calculated the observed directional difference between the empirical networks for specialization (*H₂’*) and modularity (*DIRTLPAwb*+ algorithm), defined as the index value of the focal network minus that of the comparison network (e.g., nocturnal − diurnal). Positive values therefore indicated higher specialization or modularity in the focal network. Next, using the *r2dtable* algorithm, we generated 1,000 pairs of null networks while keeping the marginal totals of each respective interaction matrix fixed. For each pair of null networks, we calculated the corresponding network-level index and its directional difference, generating a null distribution of expected differences. We then compared the observed directional difference between each pair of empirical networks with the distribution of differences obtained from the paired null networks. We assessed significance using a Monte Carlo permutation test, calculated as the proportion of randomized differences greater than or equal to the observed difference when testing whether the focal network was more specialized or modular than the comparison network (adapted from Muylaert and Dodonov 2016; Anjos et al. 2018). We used a significance level of 5% (*p* ≤ 0.05). We performed all network analyses using the bipartite package (Dormann et al. 2008), version 2.23, in R (R Core Team 2026).

## Results

Across the diurnal and nocturnal plant–herbivore networks, we recorded 179 herbivorous insects from 39 species interacting with 24 plant species (71 unique interactions) from 15 families (Table S1and S2). Diurnal networks included 28 species (44% of the total), comprising 84 herbivorous insects (47%) from 15 morphospecies (38%) interacting with 13 plant species (54%) belonging to nine families (27 interactions). In contrast, nocturnal networks included 57 species (90% of the total), with 95 herbivorous insects (53%) from 34 morphospecies (87%) interacting with 23 plant species (96%) across 14 families (49 interactions).

**Fig. 1:**
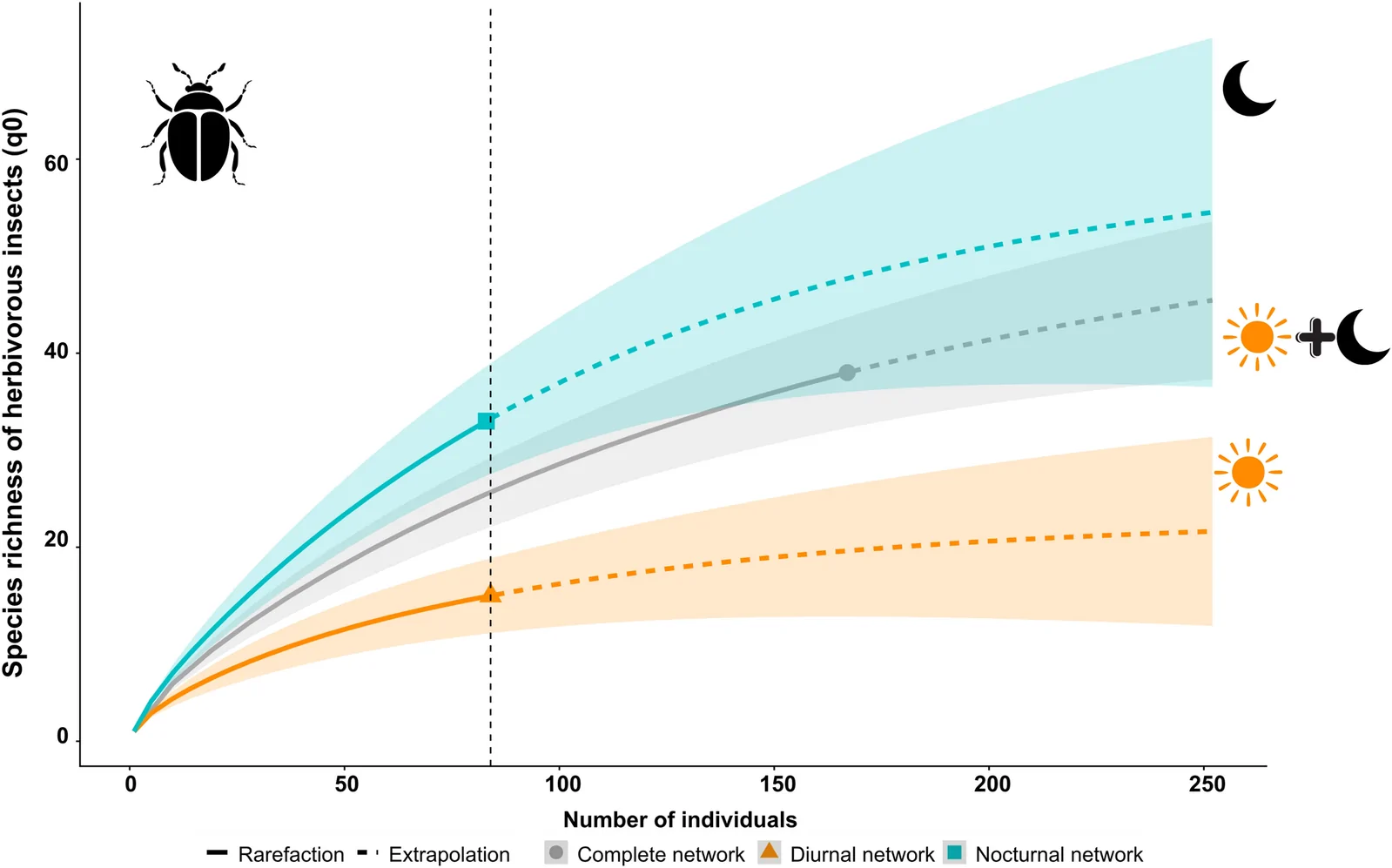
Insect herbivore diversity in the plant–herbivore networks in the Cerrado across daytime periods. Blue corresponds to the Nocturnal network, orange to the Diurnal network and gray to the Complete (diurnal+nocturnal) network. Diversity patterns are based on Hill numbers for cumulative species richness. Solid lines represent interpolation, dashed lines represent extrapolation, and shaded areas indicate 95% confidence intervals

We observed that herbivore richness tended to increase with sampling effort (i.e., the number of individuals collected) across the complete, diurnal, and nocturnal networks. Nevertheless, herbivore richness did not differ between the complete and nocturnal networks. In contrast, both networks had higher richness than the diurnal network, which had approximately twice as many species (Fig. 1).

We observed a 67% dissimilarity between the diurnal and nocturnal networks (βwn = 0.676), of which 56% was due to rewiring of interactions between periods (βo = 0.383), compared with 43% from species turnover (βst = 0.293). In addition, the diurnal and complete networks differed by 33% (βwn = 0.330): 49% of this dissimilarity was due to rewiring in plant–herbivore interactions (βo = 0.163), and 50% resulted from herbivore species turnover (βst = 0.167). In contrast, the nocturnal network showed 33% dissimilarity relative to the complete network (βwn = 0.336), of which 91% was attributable to interaction rewiring (βo = 0.308) and 8% to herbivore species turnover (βst = 0.028).

**Fig. 2:**
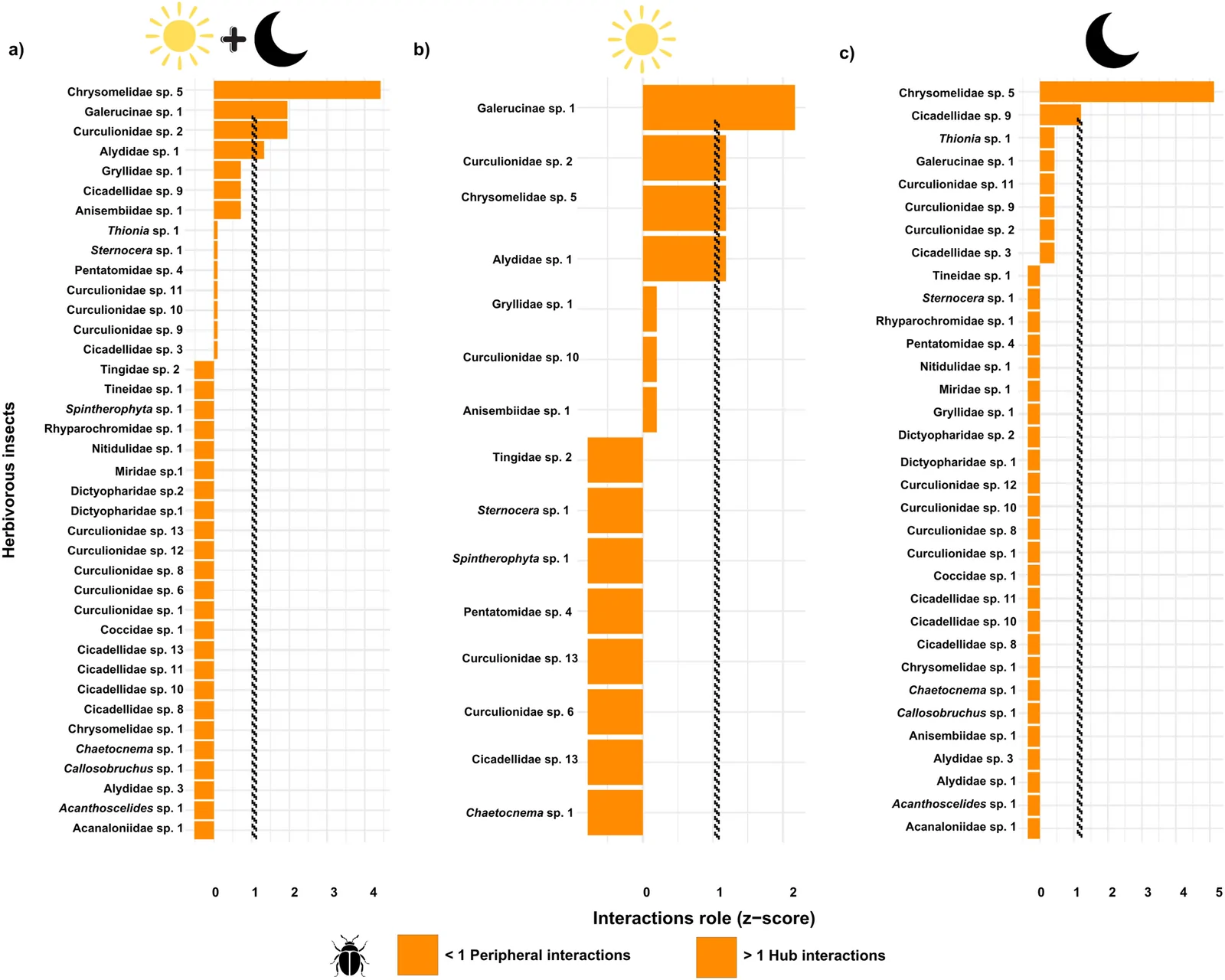
The roles of herbivorous insect species as hubs (highly connected) or peripheries (less connected) in the complete **(a)**, diurnal **(b)**, and nocturnal **(c)** plant–herbivore networks in a Brazilian Neotropical savanna (Cerrado sensu stricto*)*. The complete network is formed by combining diurnal and nocturnal networks. Herbivore hubs exhibit interaction values above 1 (black vertical line), whereas peripheral species exhibit values below 1

**Fig. 3:**
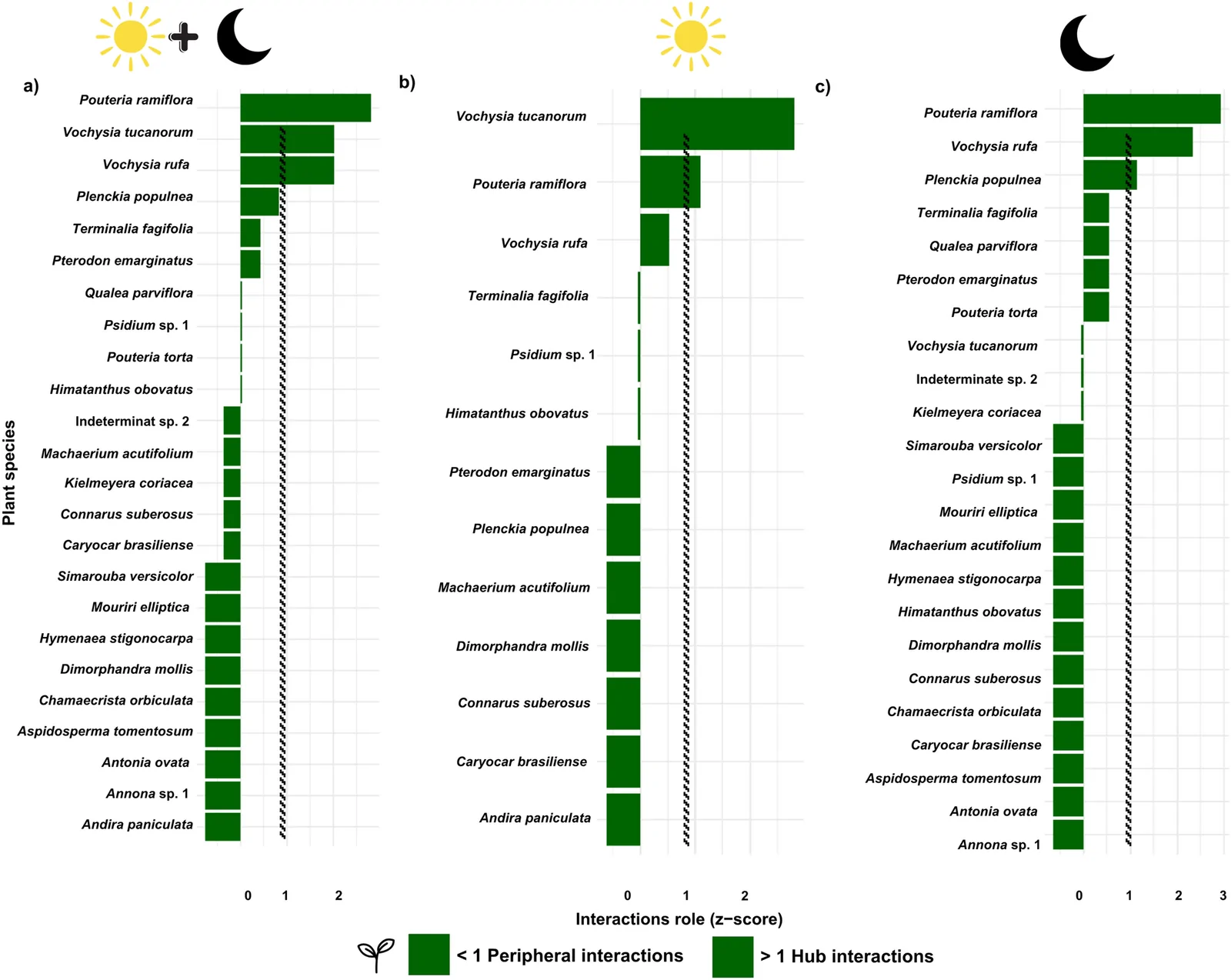
The roles of plant species as hubs (highly connected) or peripheries (less connected) in the complete **(a)**, diurnal **(b)**, and nocturnal **(c)** plant–herbivore networks in a Brazilian Neotropical savanna (Cerrado sensu stricto). The complete network is formed by combining diurnal and nocturnal networks. Plant hubs exhibit interaction values above 1 (black vertical line), whereas peripheral species exhibit values below 1

Species roles changed across periods (Tables S3 and S4). Six species were hubs in the diurnal network: four herbivorous insects (Fig. 2) and two plant species (Fig. 3). In contrast, the nocturnal network had five hubs: two herbivorous insects and three plant species. Just one hub insect was common between networks (Chrysomelidae sp. 5), with one peripheral insect in the diurnal network acting as a nocturnal hub (Cicadellidae sp. 9), and two insects changed from a diurnal hub to a nocturnal peripheral (Galerucinae sp. 1 and Alydidae sp. 1). Additionally, one plant species (*Vochysia tucanorum*) changed from hub to peripheral from day to night, with one plant species changing from peripheral to core from day to night (*Plenckia populnea*). Only one plant species was a common hub between networks (*Pouteria ramiflora*). Seven species served as hubs in the complete network: four herbivorous insects and three plant species.

**Fig. 4:**
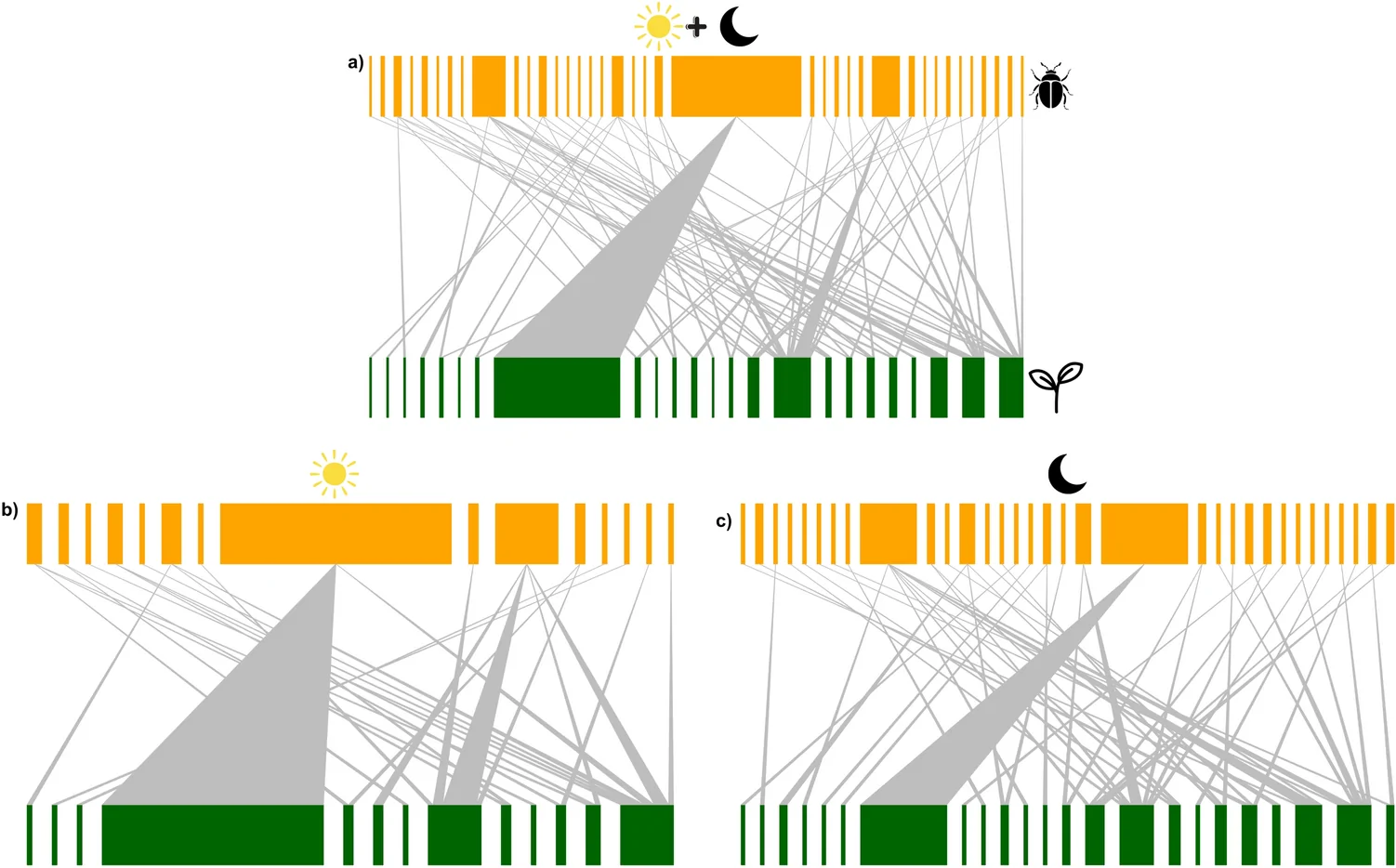
Plant–herbivore networks for the complete **(a)**, diurnal **(b)**, and nocturnal **(c)** periods in the Brazilian Neotropical Savanna (Cerrado sensu stricto). Herbivores are shown as orange rectangles (top), plants as green rectangles (bottom), and their interactions as grey links connecting both groups. Link width reflects the number of interactions recorded between each specific herbivore–plant pair

The overall network structural pattern remained consistent across periods: it was more specialized and modular than expected by chance, with a weakly nested structure. (Fig. 4; Table 1). In this way, we observed similar specialization and modularity between nighttime and daytime networks (H₂ difference = − 0.065; *p* = 0.86 / M difference = 0.225; *p* = 0.90), daytime and complete networks (H₂ = 0.083; *p* = 0.08 / M = 0.10; *p* = 0.20), and nighttime and complete networks (H₂ = 0.017; *p* = 0.49 / M = 0.12; *p* = 0.99) (Figure S1).

**Table 1:** Network descriptors observed for the plant–herbivore networks during the diurnal and nocturnal periods, as well as for the complete network (combining both periods), in a Brazilian Neotropical savanna (Cerrado sensu stricto). All descriptors were compared against null models

Network period	Network metrics	Observed value	Null model value (mean)	*p*
Complete (Day+Night)	Specialization	0.687	0.13	**<0.001**
	Modularity	0.632	0.294	**<0.001**
	Nestedness	3.718	16.169	**<0.001**
Diurnal	Specialization	0.770	0.143	**<0.001**
	Modularity	0.524	0.217	**<0.001**
	Nestedness	9.198	26.775	**<0.001**
Nocturnal	Specialization	0.705	0.151	**<0.001**
	Modularity	0.746	0.482	**<0.001**
	Nestedness	2.059	7.811	**<0.001**

Bold values in the *p* column indicate significance at *p* < 0.05

## Discussion

Herbivorous insects use vegetation in fundamental ways that shape ecosystem dynamics and support the conservation of both herbivores and their host plants (Maron and Crone 2006; Agrawal and Maron 2022). In this context, assessing how plants and herbivores interact and how environmental filters modulate those interactions are essential to understanding savanna ecosystem functioning. Because of their higher species richness, herbivorous insects were more concentrated in specific sites at night than during the day, and inclusive nighttime richness did not differ from that of the complete network. In addition, we observed both species turnover between daytime and nighttime networks and a reorganization of plant–herbivore interactions across periods. Changes in the roles of both herbivores and plants within each network accompanied these shifts. Notably, network structure remained specialized and modular with low nestedness across periods. Together, these results highlight how temporal variation shapes the ecological dynamics of plant–herbivore interactions in the Cerrado, while also revealing consistent properties across day and night periods and advancing our understanding of animal–plant interactions in this biome.

Overall, habitat structure mediates herbivorous insect occurrence in the Cerrado, which ecological factors in turn influence (Evangelista et al. 2022; Oliveira et al. 2025). For example, microclimate variations or resource availability and composition are key factors in insect behavior (Freitas et al. 2023): plant nutritional content and defensive traits shape host selection by herbivorous insects, favoring more palatable and less toxic plants (Basset 1992; Rowen and Kaplan 2016), while predators, climatic conditions, and interspecific competition further influence patterns of resource use (Bale et al. 2002; Vidal and Murphy 2018; Bird et al. 2019; Barnes and Murphy 2023). Therefore, because the sampled habitats exhibit consistent structural characteristics across periods, the higher richness of herbivorous insects observed in vegetation at night than during the day suggests that changes in temporal filters are driving this shift. Consequently, in contrast to what has been observed for the abundance of terrestrial insects in savannas, being higher during the day (Wong and Didham 2024), our findings indicate that temporal variation throughout the day is an important factor shaping herbivore species occurrence, with nighttime associated with higher species richness in the Neotropical savanna we sampled.

Consistent with differences in species richness, we observed high dissimilarity between diurnal and nocturnal networks, with herbivore turnover and rewiring of interactions acting as equally influential mechanisms, corroborating our first hypothesis. Free-living herbivorous insects exhibit low spatial specificity within the same habitat, indicating a high capacity for environmental dispersal (Novotny et al. 2009) and leading to higher beta-diversity (Lepesqueur et al. 2018). Thus, the day–night transition offers an additional window into herbivore activity in the Cerrado, with nighttime playing a key role in insect behavior. For instance, since herbivore richness was higher at night, the dissimilarity between the nighttime and daytime networks reflects restrictions on species occurrence and interactions, especially during the day. This may be demonstrated by the greater dissimilarity between the diurnal and complete networks than between the nocturnal and complete networks, which are more similar, reinforcing the idea that most species and interactions occurred at night. In other words, as observed in ant-plant networks (Luna et al. 2018), new herbivorous insects in our studied Cerrado are more likely to interact with plants at night, while even species shared between both periods interact with different plants over time. These restrictions reveal constraints on herbivorous species’ behavior, suggesting high activity specificity in each period. In this context, different groups of terrestrial insects exhibit contrasting activity patterns across the day in savannas, with some groups more abundant during the day and others at night (Wong and Didham 2024), which may help explain the observed differences in species and their interactions.

Despite similar numbers of core species across periods, consistent with our second hypothesis, the roles of herbivorous insects and plants differed between daytime and nighttime, highlighting the dynamic nature of species activity throughout the day. The shifts in species from peripheral to central positions (or vice versa) over time suggest temporal variability in interspecific interactions, ultimately altering species’ importance as hubs in herbivory-plant networks. In essence, a species’ position within the network is tied to its function: hubs act as connectors that contribute to the formation and maintenance of the network structure, while peripheral species represent the most vulnerable links and yet enhance network diversity (Dunne et al. 2002). Therefore, our results reflect behavioral shifts in partner selection between day and night, with distinct sets of insects and plants forming the network core at different periods. Because specialists and generalists play distinct roles in the ecological functioning of networks (Morrison and Dirzo 2020), temporal variation in species roles within plant–herbivore networks in the Cerrado is particularly relevant given the complementary dynamics of diurnal and nocturnal species.

The diurnal and nocturnal networks, as well as the complete network, showed a specialized structural pattern: all networks had high specialization, well-defined modularity, and low nestedness, supporting our third hypothesis. However, contrary to our fourth hypothesis, the nighttime network showed similar specialization and modularity to the daytime networks. These results indicate that underlying temporal changes across the day in our sampling savanna do not alter overall interaction patterns in plant–herbivore networks, even with shifts in species richness, composition, and roles between daytime and nighttime (see also Dáttilo et al. 2014). Our findings may relate to the observed high species turnover, leading to functionally similar interactions that contribute to the structural consistency of plant–herbivore networks in the Cerrado over time (Martins et al. 2020). For example, distinct herbivorous insects have partitioning foraging patterns (temporal, spatial, or resource focal) (Carvalho-Sposito et al. 2022; Dickie and Serrouya 2022; Sousa-Lopes and Del-Claro 2025) that facilitate coexistence and maximize fitness across species (Augustyn et al. 2016). Nevertheless, even with high partitioning, herbivorous species may exhibit functional redundancy through their interactions and maintain network patterns (Sanders et al. 2018). This structural consistency can buffer temporal changes in species composition, resulting in remarkably similar network topology between networks, consistent with previous findings from our study area, where increased plant functional diversity reduced herbivore richness, but network structure remained stable despite these changes (Freitas et al. 2026). Therefore, high turnover of species and their interactions may have increased the likelihood of functional redundancy between daytime and nighttime networks, thereby contributing to the structural consistency across periods (see also Wang et al. 2020). Even so, the consistency of structural patterns reinforces that herbivorous insects are specialists that interact with a set of correlated plant species (Fointaine and Dajoz 2009; Queiroz et al. 2013), indicating that these herbivores are restricted to interaction modules regardless of time of day.

We acknowledge that our sampling design can have introduced a methodological bias, whereby daytime vegetation beating could have temporarily altered herbivore–plant interactions and, consequently, influenced the structure of nocturnal networks. However, the patterns observed in our data suggest that any such effect was likely limited. If daytime beating had substantially displaced herbivores from the plots and affected subsequent nocturnal observations, we would expect a marked reduction in herbivore richness and greater changes in network structure. Instead, we observed higher herbivore richness at night, while network specialization structure remained relatively consistent. Although we detected changes in herbivore composition and their interactions between daytime and nighttime sampling, these changes did not substantially alter the overall structural properties of the networks. Therefore, we acknowledge that daytime beating may have introduced a short-term disturbance that could affect individual herbivore activity, but the observed patterns suggest that any such effect was likely temporary. Despite these potential short-term effects, nocturnal networks maintained structural properties broadly comparable to those observed during the day. Therefore, our results should be interpreted in light of this sampling limitation. Future studies should evaluate different sampling plots across sampling periods to further assess the temporal dynamics of herbivore–plant interactions and minimize potential carryover effects between daytime and nighttime sampling.

In conclusion, temporal variation across daily periods in our sampled Neotropical savannas is a key mechanism driving ecological differentiation among herbivorous insects and their host plants, ultimately allowing shifts in species composition and in the configuration of their interactions between daytime and nighttime networks. However, although nighttime is important for the occurrence and roles of different herbivorous species relative to daytime, the mechanisms underlying the reconfiguration of species roles are not strong enough to broadly alter plant–herbivore network patterns. Consequently, these findings reinforce the need for broader studies about daily periods – such as interaction redundancies—to fully understand how different groups of free-living herbivorous insects use their habitats and resources, which is crucial for the understanding and conservation of ecosystem functioning.

## Supplementary Information

Below is the link to the electronic supplementary material.

Supplementary file1 (PDF 419 KB)

## Data Availability

The datasets used and/or analyzed during the current study are available from the corresponding author upon reasonable request.
