# Supplementary material for: The dark side of herbivorous insects in Neotropical savannas: plant–herbivore networks across diurnal and nocturnal periods
Source: Oecologia. 2026 Oct 27;208(11):161. doi: 10.1007/s00442-026-05996-1 (PMC13714073; doi:10.1007/s00442-026-05996-1)
Supplement: Supplementary file 1 [file 442_2026_5996_MOESM1_ESM.pdf]

The dark side of herbivorous insects in Neotropical savannas: plant-herbivore networks across diurnal and nocturnal periods

Jefferson Bruno B. Souza-Oliveira[1], Leonardo V. A. Costa[1], Wesley Dáttilo[2, 3], Walter S. Araújo[1]

[1] Laboratório de Interações Ecológicas e Biodiversidade, Programa de Pós-Graduação em Biodiversidade e Uso dos Recursos Naturais, Departamento de Biologia Geral, Centro de Ciências Biológicas e da Saúde, Universidade Estadual de Montes Claros, Montes Claros, MG, Brazil.

[2] Red de Ecoetología, Instituto de Ecología AC, Xalapa, Veracruz, Mexico

[3] Laboratorio Nacional de Biología del Cambio Climático, Secretaría de Ciencia, Humanidades, Tecnología e Innovación, Mexico City, Mexico

ORCID: Oliveira JBBS: 0000-0002-5392-6080; Costa LVA: 0009-0003-2539-4261; Dáttilo W: 0000-0002-4758-4379; Araújo WS: 0000-0003-0157-6151

Correspondence author: Jefferson Bruno B. S. Oliveira - jeffersonbrunoxd@gmail.com

Supplementary information

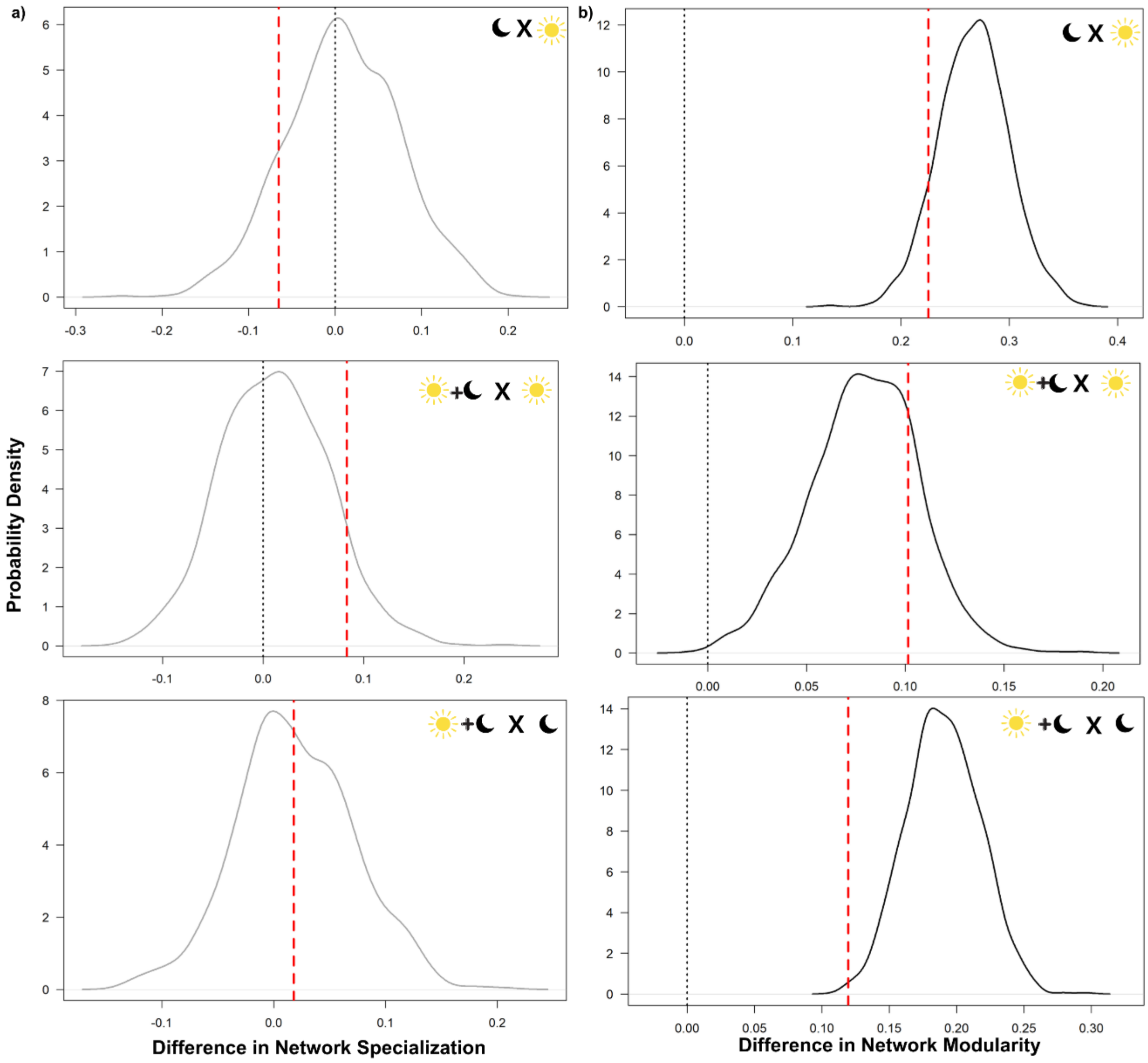

Fig. 1 Distribution of null-model differences in a) specialization (H2) and b) modularity (Q) between nocturnal, diurnal and complete networks. The red dashed line indicates the observed difference in specialization and modularity (index of network 1 − index of network 2), whereas the black dotted line indicates zero difference (similarity between networks). The black curve represents the distribution of differences produced by the null model. Positive values: network 1 is more specialized and modular than network 2. Negative values: network 1 is more modular than network 2.

Table S1 Exophagous adult herbivorous insects recorded on plants in a Neotropical savanna during the daytime (Brazilian Cerrado). The dataset includes the identification of

herbivorous insects, their host plants, and the abundance at which each insect was recorded on its respective host plant

**Plant species**	**Herbivore**	**Period**	**Abundance**
Agonandra brasiliensis Miers ex Benth. & Hook.f.	Cicadellidae sp. 11	Daytime	1
Andira paniculata Benth	Curcolionidae sp. 2	Daytime	1
Antônia ovata Pohl	*Buprestis* sp. 1	Daytime	2
Caryocar brasiliense Cambess	Galerucinae sp. 1	Daytime	1
	Spintherophyta sp. 1	Daytime	1
Connarus suberosus Planch.	Pentatomidae sp. 4	Daytime	1
Dimorphandra mollis Benth.	Curcolionidae sp. 10	Daytime	47
*Himatanthus obovatus (*Muell.Arg.)	Alydidae sp. 1	Daytime	1
Woodson	Gryllidae sp. 1	Daytime	1
Machaerium acutifolium Vogel	Galerucinae sp. 1	Daytime	2
Mouriri elliptica Mart.	Curcolionidae sp. 9	Daytime	1
	Gryllidae sp. 1	Daytime	1
Plenckia populnea Reissek	Anisembiidae sp. 1	Daytime	1
Pouteria torta (Mart.) Radlk.	Curcolionidae sp. 6	Daytime	1
	Curcolionidae sp. 13	Daytime	2
	Galerucinae sp. 1	Daytime	9
	Gryllidae sp. 1	Daytime	1
Psidium L. sp. 1	Anisembiidae sp. 1	Daytime	1
	Curcolionidae sp. 2	Daytime	1
Pterodon emarginatus Vogel	Alydidae sp. 1	Daytime	1

Qualea parviflora Mart.	Tingidae sp. 1	Daytime	1
Terminalia fagifolia Mart.	Chrysomelidae sp. 5	Daytime	2
	Curcolionidae sp. 4	Daytime	1
	Curcolionidae sp. 9	Daytime	1
	Sternocera sp. 1	Daytime	1
Vochysia rufa (Spreng.) Mart.	Alydidae sp. 1	Daytime	1
	Chaetocnema sp. 1	Daytime	1
	Chrysomelidae sp. 5	Daytime	1
	Galerucinae sp. 1	Daytime	1
Vochysia tucanorum Mart.	*Chaetocnema* sp. 1	Daytime	2
	Chrysomelidae sp. 5	Daytime	1
	Cicadellidae sp. 13	Daytime	1
	Curcolionidae sp. 2	Daytime	2
	Curcolionidae sp. 10	Daytime	2
	Galerucinae sp. 1	Daytime	3
	Tingidae sp. 2	Daytime	1

Table S2 Exophagous adult herbivorous insects recorded on plants in a Neotropical savanna during the nighttime (Brazilian Cerrado). The dataset includes the identification of

herbivorous insects, their host plants, and the abundance at which each insect was recorded on its respective host plant

**Plant species**	**Herbivore**	**Period**	**Abundance**
Annona L. sp. 1	Coccidae sp. 1	Nighttime	1
Antônia ovata Pohl.	Alydidae sp. 1	Nighttime	1
Aspidosperma tomentosum Mart.	Cicadellidae sp. 9	Nighttime	2
Caryocar brasiliense Cambess.	Chrysomelidae sp. 5	Nighttime	1
Chamaecrista orbiculata (Benth) Irwin & Barneby	Curcolionidae sp. 1	Nighttime	1
Connarus suberosus Planch.	Curcolionidae sp. 2	Nighttime	1
Dimorphandra mollis Benth	Curcolionidae sp. 10	Nighttime	23
Himatanthus obovatus (Muell.Arg.)	Galerucinae sp. 1	Nighttime	1
Woodson	Chrysomelidae sp. 1	Nighttime	1
Kielmeyera coriacea Mart. & Zucc.	Cicadellidae sp. 9	Nighttime	1
	Curcolionidae sp. 8	Nighttime	1
Machaerium acutifolium Voge.	Chrysomelidae sp. 5	Nighttime	1
Mouriri elliptica Mart.	Curcolionidae sp. 2	Nighttime	1
Unidentified	Curcolionidae sp. 9	Nighttime	1
Plenckia populnea Reissek	Chrysomelidae sp. 5	Nighttime	1
	Gryllidae sp. 1	Nighttime	1
	Thionia sp. 1	Nighttime	1
	Tineidae sp. 1	Nighttime	2
Pouteria ramiflora (Mart.) Radlk.	Alydidae sp. 3	Nighttime	1
	Chrysomelidae sp. 5	Nighttime	1

	Cicadellidae sp. 3	Nighttime	1
	Curcolionidae sp. 9	Nighttime	3
	Curcolionidae sp. 11	Nighttime	1
	Galerucinae sp. 1	Nighttime	1
	Nitidulidae sp. 1	Nighttime	1
Pouteria torta (Mart.) Radlk.	Anisembiidae sp. 1	Nighttime	1
	Chrysomelidae sp. 5	Nighttime	1
	Cicadellidae sp. 3	Nighttime	1
Psidium L. sp. 1	Pentatomidae sp. 4	Nighttime	1
Pterodon emarginatus Vogel	Cicadellidae sp. 9	Nighttime	1
	Cicadellidae sp. 11	Nighttime	1
	Dictyopharidae sp. 1	Nighttime	1
Qualea parviflora Mart.	*Acanthoscelides* sp. 1	Nighttime	2
	Sternocera sp. 1	Nighttime	1
Simarouba versicolor A.St.-Hil.	Chrysomelidae sp. 5	Nighttime	2
Terminalia fagifolia Mart.	Acanaloniidae sp. 1	Nighttime	1
	Chrysomelidae sp. 5	Nighttime	5
	Cicadellidae sp. 8	Nighttime	1
Vochysia rufa (Spreng.) Mart.	*Callosobruchus* sp. 1	Nighttime	1
	Chrysomelidae sp. 5	Nighttime	3
	Curcolionidae sp. 12	Nighttime	1
	Dictyopharidae sp. 2	Nighttime	2
	Miridae sp. 1	Nighttime	1
	Rhyparochromidae sp. 1	Nighttime	1

Vochysia tucanorum Mart.	*Chaetocnema* sp. 1	Nighttime	1
	Thionia sp. 1	Nighttime	1

Table S3 The role of herbivorous insect species as hubs (highly connected) or peripherals (less connected) in complete (a), diurnal (b), and nocturnal (c) plant-herbivore networks in a Neotropical Cerrado, Brazil. The complete network is constructed by combining the diurnal and nocturnal networks. Herbivore hub species exhibit interaction values above 1, whereas peripheral species show values below 1. Species with negative interaction values are considered highly peripheral

**Período**	**Grupo**	**Espécie**	**Specie role**
Daytime	Herbivorous insects	Galerucinae sp. 1	2.169
		Curcolionidae sp. 2	1.183
		Chrysomelidae sp. 5	1.183
		Alydidae sp. 1	1.183
		Gryllidae sp. 1	0.197
		Curcolionidae sp. 10	0.197
		Anisembiidae sp. 1	0.197
		Tingidae sp. 2	-0.788
		Sternocera sp. 1	-0.788
		Spintherophyta sp. 1	-0.788
		Pentatomidae sp. 4	-0.788
		Curcolionidae sp. 13	-0.788
		Curcolionidae sp. 6	-0.788

		Cicadellidae sp. 13	-0.788
		Chaetocnema sp. 1	-0.788
Nighttime	Herbivorous insects	Chrysomelidae sp. 5	5.125
		Cicadellidae sp. 9	1.210
		Thionia sp. 1	0.427
		Galerucinae sp. 1	0.427
		Curcolionidae sp. 11	0.427
		Curcolionidae sp. 9	0.427
		Curcolionidae sp. 2	0.427
		Cicadellidae sp. 3	0.427
		Tineidae sp. 1	-0.355
		Sternocera sp. 1	-0.355
		Rhyparochromidae sp. 1	-0.355
		Pentatomidae sp. 4	-0.355
		Nitidulide sp. 1	-0.355
		Miridae sp. 1	-0.355
		Gryllidae sp. 1	-0.355
		Dictyopharidae sp. 2	-0.355
		Dictyopharidae sp. 1	-0.355
		Curcolionidae sp. 12	-0.355
		Curcolionidae sp. 10	-0.355
		Curcolionidae sp. 8	-0.355
		Curcolionidae sp. 1	-0.355
		Coccidae sp. 1	-0.355

		Cicadellidae sp. 11	-0.355
		Cicadellidae sp. 10	-0.355
		Cicadellidae sp. 8	-0.355
		Chrysomelidae sp. 1	-0.355
		Chaetocnema sp. 1	-0.355
		Callosobruchus sp. 1	-0.355
		Anisembiidae sp. 1	-0.355
		Alydidae sp. 3	-0.355
		Alydidae sp. 1	-0.355
		Acanthoscelides sp. 1	-0.355
		Acanaloniidae sp. 1	-0.355
Complete (Day+Night)	Herbivorous insects	Chrysomelidae sp. 5	4.419
		Galerucinae sp. 1	1.949
		Curcolionidae sp. 2	1.949
		Alydidae sp. 1	1.332
		Gryllidae sp. 1	0.714
		Cicadellidae sp. 9	0.714
		Anisembiidae sp. 1	0.714
		Thionia sp. 1	0.097
		Sternocera sp. 1	0.097
		Pentatomidae sp. 4	0.097
		Curcolionidae sp. 11	0.097
		Curcolionidae sp. 10	0.097
		Curcolionidae sp. 9	0.097

	Cicadellidae sp. 3	0.097
	Tingidae sp. 2	-0.519
	Tineidae sp. 1	-0.519
	Spintherophyta sp. 1	-0.519
	Rhyparochromidae sp. 1	-0.519
	Nitidulide sp. 1	-0.519
	Miridae sp. 1	-0.519
	Dictyopharidae sp. 2	-0.519
	Dictyopharidae sp. 1	-0.519
	Curcolionidae sp. 13	-0.519
	Curcolionidae sp. 12	-0.519
	Curcolionidae sp. 8	-0.519
	Curcolionidae sp. 6	-0.519
	Curcolionidae sp. 1	-0.519
	Coccidae sp. 1	-0.519
	Cicadellidae sp. 13	-0.519
	Cicadellidae sp. 11	-0.519
	Cicadellidae sp. 10	-0.519
	Cicadellidae sp. 8	-0.519
	Chrysomelidae sp. 1	-0.519
	Chaetocnema sp. 1	-0.519
	Callosobruchus sp. 1	-0.519
	Alydidae sp. 3	-0.519
	Acanthoscelides sp. 1	-0.519

		Acanaloniidae sp. 1	-0.519

Table S4 The role of plant species as hubs (highly connected) or peripherals (less connected) in complete (a), diurnal (b), and nocturnal (c) plant-herbivore networks in a Neotropical Cerrado, Brazil. The complete network is constructed by combining the diurnal and nocturnal networks. Plant hub species exhibit interaction values above 1, whereas peripheral species show values below 1. Species with negative interaction values are considered highly peripheral

**Período**	**Grupo**	**Espécie**	**Specie role**
Daytime	Host plants	*Vochysia tucanorum*	2.806
		Pouteria ramiflora	1.096
		Vochysia rufa	0.526
		Terminalia fagifolia	-0.043
		Psidium sp. 1	-0.043
		Himatanthus obovatus	-0.043
		Pterodon emarginatus	-0.613
		Plenckia populnea	0.613
		Machaerium acutifolium	0.613
		Dimorphandra mollis	0.613
		Connarus suberosus	0.613
		Caryocar brasiliense	0.613
		Andira paniculata	0.613
Nighttime	Host plants	*Pouteria ramiflora*	2.930

		Vochysia rufa	2.334
		Plenckia populnea	1.141
		Terminalia fagifolia	0.544
		Qualea parviflora	0.544
		Pterodon emarginatus	0.544
		Pouteria torta	0.544
		Vochysia tucanorum	-0.051
		Unidentified	-0.051
		Kielmeyera coriacea	-0.051
		Simarouba versicolor	-0.648
		Psidium sp. 1	-0.648
		Mouriri elliptica	-0.648
		Machaerium acutifolium	-0.648
		Hymenaea stigonocarpa	-0.648
		Himatanthus obovatus	-0.648
		Dimorphandra mollis	-0.648
		Connarus suberosus	-0.648
		Chamaecrista orbiculata	-0.648
		Caryocar brasiliense	-0.648
		Aspidosperma tomentosum	-0.648
		Antonia ovata	-0.648
		Annona sp. 1	-0.648
Complete	Host plants	*Pouteria ramiflora*	2.813
(Day+Night)		*Vochysia tucanorum*	2.813

Vochysia rufa	2.813
Plenckia populnea	0.827
Terminalia fagifolia	0.430
Pterodon emarginatus	0.430
Qualea parviflora	0.033
Psidium sp. 1	0.033
Pouteria torta	0.033
Himatanthus obovatus	0.033
Unidentified	-0.364
Machaerium acutifolium	-0.364
Kielmeyera coriacea	-0.364
Connarus suberosus	-0.364
Caryocar brasiliense	-0.364
Simarouba versicolor	-0.761
Mouriri elliptica	-0.761
Hymenaea stigonocarpa	-0.761
Dimorphandra mollis	-0.761
Chamaecrista orbiculata	-0.761
Aspidosperma tomentosum	-0.761
Antonia ovata	-0.761
Annona sp. 1	-0.761
Andira paniculata	-0.761
